# The Efficacy of Polyunsaturated Fatty Acids as Protectors against Calcium Oxalate Renal Stone Formation: A Review

**DOI:** 10.3390/nu12041069

**Published:** 2020-04-12

**Authors:** Allen L. Rodgers, Roswitha Siener

**Affiliations:** 1 Department of Chemistry, University of Cape Town, Cape Town 7701, South Africa; 2 University Stone Centre, Department of Urology, University Hospital of Bonn, 53127 Bonn, Germany; Roswitha.Siener@ukbonn.de

**Keywords:** renal stones, recurrence prevention of calcium kidney stones, urolithiasis, dietary polyunsaturated fatty acids, fish oil supplementation, eicosapentaenoic acid, arachidonic acid, treatment of hypercalciuria, treatment of hyperoxaluria

## Abstract

In the pathogenesis of hypercalciuria and hyperoxaluria, n-6 polyunsaturated fatty acids (PUFAs) have been implicated by virtue of their metabolic links with arachidonic acid (AA) and prostaglandin PGE_2_. Studies have also shown that n-3 PUFAs, particularly those in fish oil—eicosapentaenoic acid (EPA) and docosahexaenoic acid (DHA)—can serve as competitive substrates for AA in the n-6 series and can be incorporated into cell membrane phospholipids in the latter’s place, thereby reducing urinary excretions of calcium and oxalate. The present review interrogates several different types of study which address the question of the potential roles played by dietary PUFAs in modulating stone formation. Included among these are human trials that have investigated the effects of dietary PUFA interventions. We identified 16 such trials. Besides fish oil (EPA+DHA), other supplements such as evening primrose oil containing n-6 FAs linoleic acid (LA) and γ-linolenic acid (GLA) were tested. Urinary excretion of calcium or oxalate or both decreased in most trials. However, these decreases were most prominent in the fish oil trials. We recommend the administration of fish oil containing EPA and DHA in the management of calcium oxalate urolithiasis.

## 1. Introduction

During the past 30 years, studies have claimed that the dietary intake of n-3 polyunsaturated fatty acids, particularly those occurring in marine fish and fish oils, reduces the risk of urinary stone formation [1,2,3,4,5,6,7]. Other studies have shown an increased risk associated with diets rich in the n-6 fatty acid arachidonic acid (AA) [1,3].

Studies that have suggested a protective effect for n-3 fatty acids have demonstrated reductions in important stone risk factors such as hypercalciuria [2,4,8,9,10], hyperoxaluria [6], or both [1,7,11]. On the other hand, other studies have questioned the veracity of this association [8,12,13,14,15]. For example, the study by Taylor and co-workers involving over 230,000 subjects in 3 three large national US cohorts showed that an increased intake of dietary n-3 PUFAs is highly unlikely to reduce the risk for kidney stone formation [13]. Studies suggesting a lithogenic role for n-6 PUFAs have based their claims on AA being a precursor of the pro-inflammatory and pro-aggregatory dienoic metabolite prostaglandin PGE_2_ [1], which is thought to affect calcium excretion by influencing renal tubular function and possibly by increasing intestinal calcium absorption [1,3,11]. Also, increased AA may induce hyperoxaluria by activating anion carriers and, consequently, the intestinal and renal transport of oxalate [11]. The process of renal parenchymal calcification is then triggered, which itself is likely to be etiologically significant in the pathogenesis of calcium oxalate stone formation.

In view of these contradictory findings, an assessment of the potential protective role of polyunsaturated fatty acids in stone formation and the mechanisms by which these fatty acids achieve these effects is warranted. The present review interrogates reports and studies published since the early 1950s on the various approaches employed by researchers for testing this notion and offers convincing evidence that studies involving dietary PUFA intervention are the most effective of these. Our review shows that the ingestion of fish oil, a rich source of n-3 fatty acids, reduces important physicochemical risk factors for calcium oxalate kidney stones. 

## 2. Types of Studies

This review is limited to studies involving human subjects. Animal studies have not been included. Several different approaches have been adopted in the investigation of the effects of PUFA ingestion on the risk of nephrolithiasis in humans. Approaches include stone incidence rates, baseline habitual diets, baseline urinary and plasma PUFA profiles, and the effects of PUFA dietary interventions on urinary and plasma risk factors in stone formers and healthy subjects. 

### 2.1. Population Studies of Stone Incidence

Researchers have investigated population groups in which stone incidence is rare and whose habitual dietary practices with respect to PUFA ingestion are documented. Two such population groups have been identified during the past 70 years—the Inuit peoples of the Arctic regions of Greenland, Canada, and Alaska (formerly referred to as Eskimos) and the black inhabitants of South Africa (formerly referred to as Bantu). As the following summary will indicate, it is somewhat ironic that although some of these population-based studies are founded on a self-perpetuating urban legend and other studies have reported inconclusive or counterintuitive findings, they have nevertheless generated high levels of interest which themselves have led to excellent research efforts finally culminating in the present review. 

The notion that PUFAs occurring in fish oils is protective against renal stone disease arises from the widely cited claim that urolithiasis is extremely rare in Alaskan (Canadian) and Greenland Eskimos, whose diet is predominantly rich in this food source. However, scrutiny of the mainstream literature reveals that the observation of a protective effect against kidney stones per se does not seem to have been reported. The earliest mention of stone rarity in Eskimos that we could find was reported in the paper by Modlin [16] in which he states, “Eskimos rarely form renal stones”, but he does not provide any literature references to substantiate this point. Interestingly, in a paper on the relative importance of essential fatty acids in the Eskimo diet published 14 years later, Sinclair provides a list of several diseases that are rare in Eskimos [17]. Although he includes gallstones, he does not mention renal stones. Ironically, several studies by other authors routinely cite these papers (particularly the one published by Modlin) as the authoritative references for the epidemiological anomaly in Eskimos [1,3,4,11]. Other studies also refer to the rarity of renal stones in Eskimos but mistakenly provide references which describe the rarity of heart atherosclerotic and degenerative diseases without mentioning renal stones [4,5,7,18]. What is undisputed, however, is that the habitual diet of Eskimos is rich in n-3 PUFAs [17,19,20] and that the occurrence of several degenerative diseases is extremely rare in this population group [1,17,18]. This epidemiological observation, coupled with the notion of an apparent rarity of stones, has motivated many excellent research studies to investigate the role of PUFAs in stone disease. 

The rarity of renal stones in the South African black population (B) (<1%) accompanied by a corresponding incidence rate of 10% in the white population (W) is well documented [21,22,23]. In a recent study of the FA content in the habitual diets of 10 white and 10 black South African subjects, the authors hypothesized that potential differences in the respective dietary PUFA intakes in the groups might demonstrate a protective effect in B [15]. However, their findings did not support their hypothesis as differences in the intakes of three PUFAs, important in the assessment of stone risk, and their concomitant concentrations in plasma and red blood cell total phospholipids were contrary to what might have been expected. Somewhat counterintuitively, the findings indicated a higher risk in the B group [15]. Details of the mechanisms by which PUFAs affect the risk factors for stone formation are given in the following paragraphs. 

### 2.2. Habitual PUFA Intake in Stone-Formers vs. Healthy Subjects and in High-Risk vs. Low-Risk Groups

Surprisingly, we were unable to find any studies in which baseline values for the dietary intake of PUFAs were simultaneously reported in stone formers and healthy subjects. Indeed, there was only one study in which dietary intakes were reported and these intakes were for stone-formers alone [24]. Thus, our review does not allow us to test the hypothesis that intakes in stone-formers and normals might be different and that they might be related to stone protection or formation. Similarly, the only study that compared intakes in high- and low-risk groups was that mentioned above, involving W and B South African subjects, in whom counterintuitive results were found [15]. Thus, like the population studies described above, evidence relating the habitual dietary intake of PUFAs to stone formation or protection is also lacking. 

### 2.3. Baseline Phospholipid PUFA Profiles in Stone-Formers vs. Healthy Subjects, and in High-Risk vs. Low-Risk Groups 

To test the notion that PUFAs may play a role in the prevention or pathogenesis of renal stone formation, researchers have speculated that fundamental differences might exist in PUFA profiles in stone-formers and healthy individuals. Attention has focused on n-3 FAs like alpha-linolenic acid (ALA), and EPA, and on n-6 FAs like linoleic acid (LA), gamma-linolenic acid (GLA), and arachidonic acid (AA). Baggio and co-workers tested the hypothesis that ion flux cell abnormalities (previously associated with calcium oxalate stone formation) were secondary effects to an anomaly in renal cell membrane composition [11]. They found lower content of LA and a higher concentration of AA in plasma and erythrocyte membrane phospholipids and an increased AA/LA ratio in idiopathic calcium stone-formers compared to healthy controls [11]. A higher concentration of AA in stone formers was confirmed in a later study by Baggio et al., who also reported higher PGE_2_ in this group [9]. In the same year, Messa et al. performed a study to confirm these findings and to test whether any relationship exists between the FA composition of red blood cell membranes and the main metabolic factors involved in stone formation [12]. Interestingly, in direct contradiction to the studies of Baggio et al., they reported lower AA, LA, and DHA in the red blood cell membranes of stone-formers [12]. However, they did find that hyperoxaluric stone-formers had a relatively higher AA than stone-formers with normal oxalate excretion [12]. Finally, Rodgers et al. argued that the significantly lower occurrence of stones in South Africa’s black population relative to that in the white population presented them with an ideal opportunity to test whether PUFAs might play a role in this anomaly. They examined PUFA plasma and urinary profiles in their low- and high-risk groups B and W, respectively [15,25], and reported findings that were also counterintuitive relative to the Baggio model [11]. The concentration of AA was significantly higher in the low-risk B group, and there was no difference between the groups in AA/LA ratios [15]. Thus, consideration of the findings reported in this paragraph leads to the conclusion that inter-group comparisons in PUFA profiles have not been consistent, and, as such, have not provided irrefutable evidence of their potential role in nephrolithiasis. 

### 2.4. PUFA Dietary Interventions

Numerous intervention studies have been conducted. Researchers have focused their attention on examining the effects (if any) of PUFA ingestion on the well-established physicochemical risk factors of urinary calcium, oxalate, citrate, magnesium, and phosphate [26]. A summary of these is given in Table 1. We note that a total of 16 studies is listed. PUFAs which were tested are EPA (2 studies) [1,5], EPA+DHA (8 studies) [1,2,6,7,8,9,11,14], LA+GLA (5 studies) [2,10,25], and EPA+DHA+GLA+LA (one study) [2]. These have been delivered as fish oil or as isolated supplements (EPA, GLA, evening primrose oil). 

Urinary Ca excretion decreased in 10 of these (62.5%). There was no change in this urinary parameter in the remaining 6 studies. Inspection of the composition of the test substances shows that EPA and DHA are common in 8 of the 10 studies in which urinary Ca excretion decreased, while LA and GLA (as evening primrose oil) account for the other two. 

In examining the efficacy of PUFAs for reducing urinary Ca, we need to account for the studies in which this urinary parameter did not change. In their paper on the effect of EPA, Yasui and co-workers found a reduction in stone recurrence [5]. Surprisingly urinary Ca excretion was unchanged [5]. This was in contrast an earlier study of theirs in which the same EPA supplement was used but urinary Ca decreased [4]. They suggested that this inconsistency might be due to differences in their respective patient cohorts as their earlier study involved hypercalciuric patients while their subsequent study did not [5]. Interestingly, Table 1 shows that four of the other studies in which no decrease in urinary Ca occurred used healthy subjects as their test group as opposed to stone-formers [6,10,14,25] lending support to the notion that PUFA-induced reduction of Ca excretion is possibly restricted to stone-forming patients. Indeed, Buck and co-workers commented in an earlier paper that the normalizing effect of fish oil with respect to urinary calcium and oxalate levels was more pronounced in patients in whom these parameters were markedly raised [1]. The table also shows that evening primrose oil (i.e., LA+GLA) achieved a reduction in Ca excretion in healthy subjects [10] but not in stone-formers [2]. Clearly, the number of studies involving evening primrose oil is too limited to warrant us pronouncing on its efficacy.

Table 1 shows that urinary oxalate was measured in thirteen studies after the administration of the PUFA test substance. It decreased in four of these [1,6,7,11] but remained unchanged in eight studies [2,8,10,14,25]. (Reference 2 describes three studies; reference 10 describes 2 studies; reference 25 also describes 2 studies, but an unchanged urinary oxalate was recorded in only one of them). One study found that it increased [25]. Of importance is that the supplement that was administered in the four studies in which urinary Ox decreased was fish oil. Other supplements (LA/GLA and GLA alone) [2,10,25] had no effect on Ox excretion. Fish oil failed to lower Ox excretion in three studies [2,8,14]. Rothwell et al. commented that this difference in the effect of fish oil on oxalate handling is difficult to explain but that relatively higher baseline Ox levels may respond more readily to PUFA supplementation [8]. This is in accordance with the previously mentioned comment by Buck and co-workers that the normalizing effect of fish oil is more pronounced in patients with relatively higher Ox excretion levels [1]. This was subsequently confirmed by Lange et al. [14]. 

Urinary citrate was measured in 10 studies involving different PUFAs. Five of these were performed after fish oil ingestion [2,6,7,8,14], three were performed after EPO ingestion (2,10), and two were performed after GLA ingestion [25] (references 10 and 25 each describe studies invoving two race groups, B and W). Although an increase in urinary citrate was observed in 40% of these [7,10,25] (both race groups in reference 10; W race group in reference 25), no change in this parameter occurred in any of the other studies [2,6,14,25] (B race group in reference 25) except for one study in which a decrease was observed [8]. Clearly, the effect of PUFAs on the urinary excretion of citrate is yet to be fully established, and as such, it requires further investigation in future studies. Similarly, changes in urinary Mg and Phos excretion have occurred too seldom for us to draw any firm conclusions. 

Of critical importance is that there has been only one study that has used stone recurrence as its outcome measure for assessing the efficacy of PUFAs in reducing stone formation per se [5]. In that study, 29 CaOx stone-forming patients were followed over a period of 8 years, during which a highly purified supplement of EPA was administered (1.8 g per day for three years). Stone recurrence was monitored before, during, and after supplementation. The incidence rate of nephrolithiasis was significantly lower during the administration of the supplement compared to before and after its administration. Intriguingly, no change was observed in any of the urinary parameters that were measured, including Ca, Mg, and Phos, thereby ruling out the possibility of EPA having reduced stone incidence via alterations in these urinary risk factors. Remarkably, urinary Ox was not measured in this study [5]. We can only speculate that a reduction in the urinary excretion of this component might have been a contributory factor in reducing stone incidence. Of course, the possibility exists that some factor besides urine biochemistry might have caused this effect. Future studies involving the effect of PUFA ingestion on stone recurrence will most likely resolve this puzzle. 

Consideration of the findings reported in Table 1 and described in the preceding paragraphs indicates that the PUFAs EPA and DHA (either in combination in fish oil or as individual, purified supplements) reduced urinary Ca in 66.7% and urinary oxalate in 57.1% of the studies in which they were measured. In the remainder of these studies (33.3% and 42.9%, respectively), there was no change in either parameter. Findings also indicate that a favorable outcome involving an increase in urinary citrate was observed in four studies involving different PUFAs [7,10,25] (both race groups in reference 10; W race group in reference 25). Since these findings are potentially promising for possible application in stone management, explanatory mechanisms for these effects are warranted.

## 3. Mechanisms and Metabolic Pathways

Prior to discussing the proposed mechanisms for the role of PUFAs in renal stone formation, it is helpful to briefly mention some important factors in the pathogenesis of stone formation itself. The most common stone types are calcium oxalate, calcium phosphate, and uric acid, with the first of these representing over 70% of all stones. The pathogenesis of each is complex and involves many processes that themselves rely on a multitude of factors [27]. However, common to all of them is an initial physicochemical step involving crystal formation. This process is initiated by the driving force of supersaturation [28]. It is in this area that researchers believe PUFAs may play a role. 

Because of its high occurrence rate, calcium oxalate stones have received the most attention. The most important pathophysiological factor for calcium nephrolithiasis is hypercalciuria. Calcium increases the ionic activity and saturation of crystallizing calcium salts (oxalate and phosphate). Equally important is hyperoxaluria. Because its concentration in urine is much lower than calcium, oxalate is the limiting factor for binding to calcium in this environment. The supersaturation of calcium oxalate is thus critically dependent on urinary oxalate concentration. Urinary citrate is also an important pathogenic risk factor by virtue of its ability to bind free calcium and form soluble calcium citrate, thereby reducing supersaturation of calcium-containing salts. It is thus appropriately categorized as a urinary stone inhibitor.

Epidemiological and clinical studies with respect to urinary calcium, oxalate, and citrate excretions have been described above. The paragraphs below describe the mechanisms by which these effects are thought to occur.

**Figure 1:**
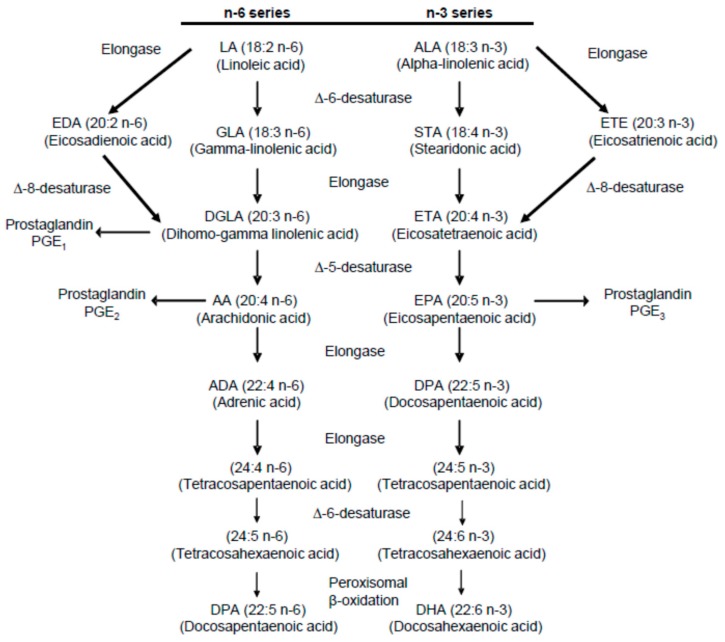
Summary of the metabolic pathway for n-6 and n-3 polyunsaturated fatty acids [15].

In order to appreciate the role played by various PUFAs in possible stone formation and prevention, it is also helpful to refer to established metabolic pathways of n-3 and n-6 metabolism. Diagrams of these pathways have been published in several papers dealing with PUFA ingestion, hypercalciuria, and hyperoxaluria [1,2,3,10,15]. One of them is presented in Figure 1 [15]. 

### 3.1. Mechanisms for Reducing Urinary Calcium Excretion

It is well established that renal Ca excretion is influenced by the n-6 dienoic metabolite PGE_2_ and that raised urinary levels of this metabolite are associated with hypercalciuria [1,3]. Buck and co-workers were among the first to point out that EPA is an inhibitor of AA, the precursor of PGE_2_ [1,2]. They and others have suggested that dietary substitution of n-6 PUFAs (which themselves are precursors of AA synthesis) by n-3 EPA (from fish oil) culminates in its incorporation into cell membrane phospholipids at the expense of AA, thereby decreasing the activity of PGE_2_ and excretion of Ca [1,2,4]. In a series of excellent papers, Baggio and co-workers proposed in some detail that idiopathic calcium nephrolithiasis patients differ from healthy controls by virtue of an abnormal composition in their phospholipid n-6 content [9,11]. A key finding of theirs was a significantly higher content of AA and a significantly lower content of LA in plasma phospholipids in idiopathic calcium stone-formers compared with healthy subjects, culminating in a higher AA/LA ratio in the former group [11]. They suggest that this anomalous n-6 composition causes an elevation in PGE_2_ production, which in turn causes hypercalciuria. It does so by facilitating calcium absorption because of its stimulation of 1α-hydroxylase and, consequently, 1,25-hydroxyvitamin D synthesis [11]. Importantly, they showed that dietary manipulation with fish oil led to a decrease in LA and AA levels with concomitant decreases in Ca and Ox excretions. In a later study, Baggio and other co-workers proposed that a higher phospholipid AA content induces an increased calciotropic hormone concentration leading to increased intestinal calcium absorption, a decreased renal tubular calcium reabsorption, and an increased calcium bone loss, all of which are known causes of hypercalciuria [9]. Their findings that reductions in AA levels and urinary Ca excretion following fish oil supplementation were accompanied by a reduction in PGE_2_ synthesis led them to suggest that the decrease in this prostaglandin might lead to activation in renal Na/K/2Cl cotransport function, thereby causing a greater renal tubular reabsorption of sodium and Ca, and a subsequently lower calcium excretion [11]. 

The mechanism by which evening primrose oil enhances the reduction in urinary Ca excretion achieved by fish oil also deserves attention. Gamma-linolenic acid (GLA) is derived from primrose oil [2]. Like EPA, it is rapidly and readily incorporated into cell membrane phospholipids instead of AA. GLA is a precursor for the monenoic series prostaglandin (PGE_1_), which has opposite properties to the pro-aggregatory and pro-inflammatory prostaglandin PGE_2_. [2]. Replacement of PGE_2_ by PGE_3_ decreases urinary Ca excretion [2]. When primrose oil (GLA) is added with fish oil (EPA), the latter also replaces AA and produces PGE_3_, which has similar effects to those of PGE_1_ [2]. The reduction of Ca excretion is therefore enhanced.

### 3.2. Mechanism for Reducing Urinary Oxalate Excretion

Elucidating the mechanism by which fish oil reduces urinary Ox has been recognized as being extremely difficult [7,8]. As mentioned in the preceding paragraph, Baggio and co-workers identified a systemic defect in phospholipid AA levels (pertaining to a raised AA/LA ratio) in idiopathic calcium stone formers [11]. In that study, they observed a direct relationship between erythrocyte membrane levels of AA and transmembrane oxalate self-exchange rates [11]. In order to propose a mechanism that explains fish oil’s ability to decrease Ox excretion, they cited studies by Gambaro et al. in which this cell anomaly was demonstrated as being associated with increased oxalate renal clearance and renal stone activity [29,30]. They argued that modulation of AA activity will modulate Ox transporter activity and reduce urinary Ox excretion. Indeed, their study showed that fish oil normalized the erythrocyte oxalate exchange and lowered calcium and oxalate urinary excretion, thereby supporting their mechanistic hypothesis [11]. As acknowledged by the authors, this is indeed merely a hypothesis. A relationship between plasma AA content and a clinical outcome or an exchange rate does not necessarily mean that AA is playing a mechanistic role. It is not impossible that some aspect of the metabolic defect in these patients increases the conversion of LA to AA, and that increased AA is a symptom rather than a cause of the disease. 

In one of the four studies in which Ox excretion decreased after administration of fish oil, the subjects were healthy individuals [6]. This finding contradicts the hypothesis of Baggio et al. that fish oil modulates a systemic defect in phospholipid AA levels in SFs, and as such, its action is limited to patients only [11]. That the effect occurs in healthy individuals bodes well for the possibility of fish oil being a prophylactic agent for hyperoxaluria.

### 3.3. Mechanisms for Increasing Urinary Citrate Excretion

In the present review, increases in urinary Cit occurred in studies after fish oil administration [7], EPO administration [10] (two race groups B and W), and administration of a GLA supplement [27].

Authors in the first of these studies did not attempt to explain how the increase in citrate excretion might have arisen [7]. However, in the latter two studies, the authors pointed out that a tricarboxylate transporter located in the inner mitochondrial membrane mediates the electroneural efflux of citrate from the mitochondrial matrix, in exchange for cytosolic malate or succinate, and that this process has been shown to be inhibited by n-3 and n-6 PUFA supplements [10]. This prevents the translocation of citrate from the mitochondria to the cytosol. Since both supplements inhibit lipogenesis, they hypothesized that when EPO or supplemental GLA is ingested, lipogenesis is inhibited, thereby reducing citrate consumption. This results in more plasma citrate being available to filter through the glomerulus, culminating in an increase in Cit excretion [10].

In the only study in which urinary Cit excretion decreased after fish oil administration, the authors did not suggest a mechanism to explain this effect [8]. No change in this urinary parameter was observed in two studies [6,25]. The authors did not comment on the absence of this effect in the former of these [6]. However, in the latter study in which a decrease in Cit excretion was observed in one race-group but not the other, a racial-based difference in the sensitivity towards the ingested LA/GLA ratio was speculated [25].

## 4. Discussion

Notwithstanding the absence of robust evidence from the population, dietary and PUFA profile studies regarding a possible protective role of PUFAs in renal stone-formation as results from dietary intervention trials are more supportive of this notion. Our review shows that a reduction in the excretion of urinary Ca or Ox or both, with a concomitant decrease in the risk of stone formation, can be expected following the administration of fish oil (or supplemental EPA) since reductions in these parameters occurred in approximately 70% and 60%, respectively, of the studies in which they were measured. Indeed, fish oil appears to be the most efficacious of the various supplements which have been administered in trials of this nature. This is impressive, given the extreme heterogeneity of the conditions under which the trials were conducted. These included, but were not limited to gender, dosage, trial duration, subject status (healthy, stone-formers), clinical status (hypercalciuric, hyperoxaluric, normocalciuric), and PUFA combinations. It is because of this heterogeneity (and the relatively small number of studies) that we elected not to perform a meta-analysis of the data.

The success of fish oil in reducing Ca excretion in stone-forming patients (and the concomitant risk of further stones) allows us to recommend its use as a therapeutic supplement in the treatment of such patients. Given that our review has also demonstrated the efficacy of fish oil for reducing oxalate excretion, it makes sense that a regimen in which it achieves decreases in these important urinary risk factors would be most desirable. Examination of the doses and administration periods in the three studies in which such decreases occurred simultaneously shows that Buck and co-workers gave 10 g daily (corresponding to 1.8 g EPA and 1.2 g DHA daily) for 8 weeks [1], Baggio and co-workers gave 2.55 g (corresponding to 1.29 g EPA and 0.99 g DHA daily) daily for 30 days) [11] and Ortiz-Alvarado and co-workers gave 1.2 g fish oil (EPA and DHA composition not given) daily for 9 months [7]. Finding a balance between dosage and duration is required for determining the optimum regimen. Based on the aforementioned observations, we suggest that a dose of about 1.3 g EPA and 1.0 g DHA as fish oil per day, as used by Baggio et al. [11], should be administered in the treatment of hypercalciuric and/or hyperoxaluric renal stone-formers, in the reasonable expectation of seeing a decrease in the urinary excretion of Ca and Ox within about one month. Dosages should be maintained permanently in patients responding to treatment with fish oil.

Our review has also shown that other PUFAs, besides those in fish oil (evening primrose oil, GLA), might have the potential to produce favorable changes in urinary Ca and citrate with respect to reducing stone risk, but importantly these have occurred in isolated cases only [10,25]. Interestingly, it has been suggested that the observation of a favorable finding in the study involving evening primrose oil [10] but not in that involving a GLA supplement [25] poses a question around the significance of the relative amounts of LA and GLA in the ingested substance [25]. Notwithstanding these interesting results, the paucity of studies involving the administration of evening primrose oil alone or in combination with fish oil, make it impossible to pronounce definitively on its efficacy as a prophylactic or therapeutic agent in the management of urinary Ca in the context of calcium nephrolithiasis. Further investigation of EPO and GLA supplements is warranted. 

Ideally, when testing any substance for its potential prophylactic and therapeutic efficacy regarding a particular pathology, the outcome measure should be occurrence and/or recurrence of the disease itself. PUFA action in calcium oxalate urolithiasis is no exception. Unfortunately, as described earlier, only one such study has been performed. Until such time that more trials of this nature are implemented, its role will remain unconfirmed. 

## Figures and Tables

**Table 1:** Human trials investigating the effect of n-3 and n-6 polyunsaturated fatty acid ingestion on renal stone formation risk factors.

	**Buck 1991 [1]**	**Buck 1994 [2]** **∗**	**Rothwell 1993 [8]**	**Baggio 1996 [11]**	**Baggio 2000 [9]**	**Yasui 2001 [4]**
**Type of trial**	Open	Double-blind, placebo-controlled				
**Diet controlled/standardized**	Yes	Yes	X	Yes	Yes	No
**No. subjects**	12	40	18	24P, 18C	20P,15C	88
**Gender**	8M, 4F	34M, 6F	male	13M, 11F	17M, 3F	67M, 21F
**Clinical classification**	Recurrent hypercalciuric/ hyperoxaluric SF	Recurrent idiopathic SF	Recurrent SF (hypercalciuric)	Recurrent idiopathic SF	Recurrent idiopathic SF	Recurrent SF
**Stone type**	X		X	CaOx	CaOx	“mainly” Ca
**Test substance (type)**	Fish oil	Four different substances	Fish oil	Fish oil	Fish oil	Highly purified EPA supplement
**Test substance (dose)**	10 g daily	(i) Fish Oil (6 g/d) (ii) Evening Primrose Oil (EPO) (6 g/d) (iii) Fish oil + EPO (6 g/d)	5 g (3x per day) = 15 g	0.85 (3xper day) = 2.55 g	0.85 (3xper day) = 2.55 g	600 mg/d (3x per day) = 1.8 g
**Equivalent PUFA dose per day**	EPA: 1.8 g DHA: 1.2 g	(i) EPA: 1.08 g DHA: 360 mg (ii) GLA: 480 mg LA: X (iii) EPA: 204 mg DHA: 132 mg GLA: 408 mg	EPA: 2.7 g DHA: 1.8 g	EPA: 1.29 g DHA: 0.99 g	EPA: 1.29 g DHA: 0.99 g	EPA: 1.8 g
**Duration of supplementation**	8 weeks	12 weeks	4 weeks	30 days	30 days	3 months,
**Type of urine collection**	24 h	24 h	24 h	24 h	Overnight fasting	24 h
**Control group**	X	Yes (sunflower oil)	X	9M, 9F	12M, 3F	X
**Urinary Ca**	decreased	(i) decreased (ii) no change (iii) decreased	decreased	decreased	decreased	Decrease in hypercalciuric but not normocalciuric patients
**Urinary Ox**	decreased	No change	No change	decreased	X	X
**Urinary Cit**	X	No change	decreased	X	X	X
**Urinary Mg**	X	No change	decreased	X	X	X
**Urinary Phos**	No change	No change	decreased	X	X	Decreased after long term
**Stone recurrence**	X	X	X	X	X	X
**Other**	X	No change in serum biochemical parameters		Plasma phospholipid content of LA, AA decreased; EPA DHA increased	Plasma phospholipid content of LA, AA decreased; EPA DHA increased	Plasma phospholipid content decreased in hypercalciuric patients after long term.
	**Yasui 2008 [5]**	**Rodgers 2009 [10]** **∗∗**	**Siener 2011 [6]**	**Omar 2012 [7]**	**Lange 2014 [14]**	**Rodgers 2018 [25]** **∗∗**
**Type of trial**		Inter-race				Inter-race
**Diet controlled/standardized**	No		Yes (control phase + 3 different phases)	phytate-rich diet recommended	Yes (oxalate restricted)	No
**No. subjects**	29	8W 8B	15	29	15	10W 10B
**Gender**	22M, 7F	male	8M, 7F	15M, 14F	8M, 7F	male
**Clinical classification**	SF	Healthy	Healthy	Idiopathic SF	Healthy	Healthy
**Stone type**	CaOx	Not applicable	Not applicable	Ca	Not applicable	Not applicable
**Test substance** **(type)**	Highly purified EPA supplement	Evening primrose oil	Fish oil	Fish oil	Fish oil	GLA supplement
**Test substance (dose)**	600 mg (3xper day)	1000 mg (1 per day)	1 capsule (3xper day)	1200 mg/d	2 capsules per day	2 capsules per day
**Equivalent PUFA dose per day**	EPA: 1800 mg	LA: 720 mg GLA: 80 mg	EPA: 900 mg DHA: 600 mg	EPA: X DHA: X	EPA: 1300 mg DHA:900 mg	LA: 225 × 2 = 450 mg/d GLA: 150 × 2 = 330 mg/d
**Duration of supplementation**	36 months	20 days	30 days	9 months	30 days	30 days
**Type of urine collection**	24 h	24 h	24 h	24 h	24 h	24 h
**Control group**	X	X	X	X	X	X
**Urinary Ca**	No change	W: decreased B: decreased	No change	decreased	No change	W: no change B: no change
**Urinary Ox**	X	No change	decreased	decreased	No change	W: increased B; no change
**Urinary Cit**	X	W: increased B: increased	No change	increased	No change	W: increased B: no change
**Urinary Mg**	No change	W: no change B: decreased	No change	X	No change	W: no change B: increase
**Urinary Phos**	No change	X	No change	X	No change	W: no change B: no change
**Stone recurrence**	Significantly lower	X	X	X	X	X
**Other**			No change in urinary PGE_2_. SS CaOx decreased	SS CaOx and CaP decreased	No change in the Tiselius risk index for CaOx	W: K increased, iCa decreased B: urate increased

∗: Three different test substances; P: patients; C: controls; ∗∗: two different race groups; X: not applicable or not measured or not reported; SS: supersaturation.
